# Antimicrobial therapy combined with C-C chemokine receptor type 2 modulation dampens mycobacteria-aggravated monocyte activation and atherosclerosis

**DOI:** 10.3389/fcvm.2026.1753414

**Published:** 2026-03-31

**Authors:** Rocio Egoavil-Espejo, April Haller, Manuel G. Feria, Sanjay K. Jain, Medha Singh, Bin Zhang, Joseph E. Qualls, David Y. Hui, Moises A. Huaman

**Affiliations:** 1 Department of Internal Medicine, University of Cincinnati College of Medicine, Cincinnati, OH, United States; 2 Department of Pediatrics, Cincinnati Children's Hospital Medical Center, Cincinnati, OH, United States; 3 Cincinnati Children's Center for Molecular Imaging and Precision Medicine, Cincinnati Children's Hospital Medical Center, Cincinnati, OH, United States; 4 Department of Pediatrics, University of Cincinnati, Cincinnati, OH, United States; 5 Division of Biostatistics and Epidemiology, Cincinnati Children’s Hospital Medical Center, Cincinnati, OH, United States; 6 Department of Biological Sciences, Thomas More University, Crestview Hills, KY, United States; 7 Department of Pathology, University of Cincinnati College of Medicine, Cincinnati, OH, United States

**Keywords:** atherosclerosis, cardiovascular, CCR2, mycobacteria, tuberculosis

## Abstract

**Background:**

Tuberculosis is associated with increased risk of cardiovascular events. We investigated the impact of antimicrobial therapy alone and in combination with anti-CCR2 modulation in monocyte profiling and atherosclerosis development.

**Methods:**

Twelve-week-old low-density lipoprotein receptor knockout (*Ldlr*^−/−^) mice were infected with *Mycobacterium bovis* Bacille-Calmette-Guérin (BCG; 1.0–2.5 × 10^6^ colony-forming units) via the intranasal route and fed a western-type high-fat diet for 16 weeks. Mice were treated with oral isoniazid and rifampin (INH/RIF) between weeks 4 and 12 to induce microbiologic clearance, with and without intraperitoneal injections of anti-CCR2 monoclonal antibodies administered twice weekly. Age-matched infected and uninfected *Ldlr*^−/−^ mice served as controls. We assessed monocyte phenotyping using flow cytometry, and quantified atherosclerosis in aortas using Oil-Red-O staining. Plaque composition was assessed in aortic roots.

**Results:**

Compared to uninfected mice, untreated BCG-infected mice and BCG-infected mice treated with INH/RIF exhibited an expansion of Ly6C^low^ non-classical monocytes, as well as increased expression of monocyte activation markers. BCG-infected mice developed increased atherosclerotic lesions in their aortae, regardless of INH/RIF treatment. The addition of anti-CCR2 adjunctive therapy to INH/RIF treatment decreased monocyte activation markers including MHC-II, CD64, CD36, CX3CR1, and diminished interleukin-6 production upon lipopolysaccharide stimulation. Finally, anti-CCR2 adjunctive therapy decreased atherosclerosis lesions of BCG-infected INH/RIF-treated mice, while also decreasing plaque size and lipid content.

**Conclusions:**

Monocyte activation and atherosclerosis burden remained elevated after BCG clearance with antimicrobials. The addition of anti-CCR2 to antimicrobial therapy dampened monocyte activation and atherosclerosis development. Our results indicate that combining antimicrobials with CCR2 immunomodulation may reduce mycobacteria-aggravated atherosclerosis.

## Introduction

Cardiovascular diseases are the number one cause of mortality worldwide, leading to ∼19 million deaths each year (1). Besides traditional cardiovascular risk factors such as hypertension, diabetes, and hyperlipidemia, growing evidence indicates that prevalent infections can be important contributors of cardiovascular risk (2–5). Specifically, studies have shown that a globally common infection such as tuberculosis is associated with a two-to-three-fold increased risk of developing atherosclerotic cardiovascular events (6, 7). Even after successful tuberculosis treatment, post-tuberculosis survivors have a three-fold increased risk of long-term all-cause mortality compared to the general population, with most deaths attributable to cardiovascular events (8). It is estimated that about a quarter of the world population has been exposed to *Mycobacterium tuberculosis* (9). Furthermore, there are about 10 million new cases of active tuberculosis diagnosed each year and approximately 155 million post-tuberculosis survivors alive worldwide (10). Therefore, understanding how mycobacterial infections enhance cardiovascular risk is essential to design interventions that could mitigate such risk.

In the low-density lipoprotein receptor knockout (*Ldlr*^−/−^) mouse model of atherosclerosis, we previously demonstrated that infection with *Mycobacterium bovis* Bacille-Calmette-Guérin (BCG), an attenuated strain of the *Mycobacterium tuberculosis* complex, results in monocyte activation and aggravated atherosclerosis development (11). However, whether infection clearance with antimicrobial therapy is sufficient to impede persistent monocyte activation and accelerated atherosclerosis development is unknown. Furthermore, the potential effects of adjuvant therapies which target monocyte migration on infection-aggravated atherosclerosis are unclear. Importantly, CCR2 is a chemokine receptor highly expressed on monocytes which is potentially targetable. Prior studies in animal models have shown that complete absence of the CCR2 receptor results in decreased atherosclerosis formation via inhibition of monocyte/macrophages recruitment into vessels (12). In this study, we investigated the impact of antimicrobial therapy alone and in combination with anti-CCR2 modulation on monocyte profiling and atherosclerosis development of hyperlipidemic mice infected with BCG.

## Methods

### Mouse model and experimental design

**Figure 1:**
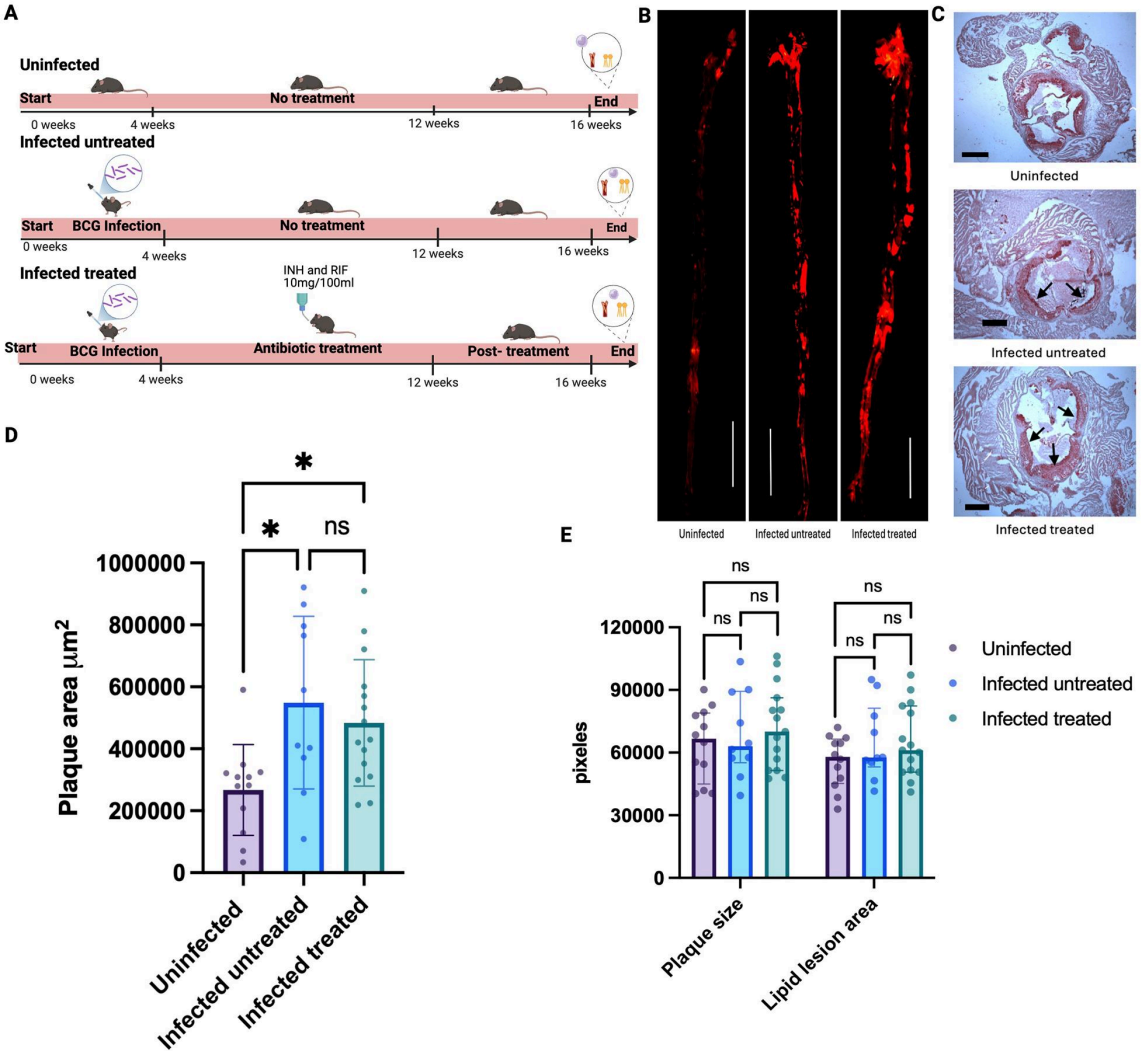
Antimicrobial therapy with rifampin and isoniazid did not reduce aggravated atherosclerosis in mice infected with *M. bovis* BCG. **(A)** Twelve-week-old male *Ldlr*^−/−^ mice were fed with western-type diet for up to 16 weeks. One randomly-selected group was used as controls (*n* = 12), while a second group was infected with *M. bovis* BCG. A group of infected mice were left untreated (*n* = 12), and the other group was treated with oral isoniazid and rifampin (INH/RIF) between weeks 4 and 12 to clear the mycobacterial infection (*n* = 16). Created in BioRender. Egoavil, R. (2026) https://BioRender.com/x7ozkgp. **(B)** Data representative of the aortae of one uninfected mouse, one BCG-infected mouse, and one BCG-infected mouse treated with INH/RIF. Images were processed by ImageJ, with a magnification of 500 μm 10X **(C)** Representative microscopic views of aortic root sections stained with Oil Red O, from one uninfected mouse, one BCG-infected mouse, and one BCG-infected mouse treated with INH/RIF, with a magnification of 500 μm 4X 1 × 1 scale. **(D)** Atherosclerotic lesions in *en face* aorta were examined using Oil Red O staining at week 16 and the plaque area was quantified in µm^2^. **(E)** Plaque size and lipid lesion area in aorta were assessed in the aortic roots using Oil Red O staining. The experiment was independently performed twice and the data were combined. Each point represents the results in each mouse*.* Two mice from the infected untreated and 1 mouse from the infected treated were excluded. *P* values were the result of Kruskal–Wallis tests for multi-group analysis and Dunn's test for pairwise comparisons; *p*-values were adjusted for multiple comparisons; ns = no significant (*p* > 0.05); *p ≤ 0.05; ** *p* < 0.01; *** *p* < 0.001; **** *p* < 0.0001.

Twelve-week-old *Ldlr*^−/−^ mice were purchased from the Jackson Laboratory and housed at the Laboratory Animal Medical Services (LAMS) within the University of Cincinnati. Mice were anesthetized and inoculated with *Mycobacterium bovis* Bacille-Calmette-Guérin (BCG) (1.0–2.5 × 10^6^ colony-forming units [CFU]) via the intranasal route as previously described (11). Upon infection, mice were fed a western-type diet containing 21% fat and 0.2% cholesterol (Envigo TD.88137 diet) for 16 weeks (Figure 1A). After 4 weeks of BCG inoculation, which is sufficient time for the mycobacterial infection to establish (13), mice received antimicrobial therapy consisting of 10 mg isoniazid (INH; Sigma-Aldrich®) plus 10 mg rifampin (RIF; Sigma-Aldrich®) per 100 mL drinking water for 8 weeks (experimental weeks 4 to 12), as previously described (14). Pyrazinamide was not included in the antimicrobial regimen as *M. bovis* is known to be intrinsically resistant to this drug (15). Mice were euthanized at 16 weeks (4 weeks after completion of INH/RIF antimicrobial therapy), placed in an induction chamber with isoflurane on a cotton gauze at a 1%–5% dose by inhalation to effect until unconsciousness. Exsanguination was performed and then death was confirmed by performing a bilateral thoracotomy: incision of the chest cavity to cause the lungs to collapse and removal of vital organs. Blood and tissues were harvested for monocyte profiling, atherosclerosis assessments, and mycobacterial cultures. Age-matched infected and uninfected *Ldlr*^−/−^ mice served as controls. We used simple randomization to assign mice to the groups. Allocation was concealed; changes on group allocation were not allowed after the randomization. Severe bullying/stress (e.g., requiring individual housing) and severe dermatitis (which can occur in hyperlipidemic mice with *Ldlr*^−/−^ genetic background) were pre-specified exclusion criteria as these conditions can affect inflammatory responses and atherosclerosis outcomes.

We then studied the effects of CCR2 immunomodulation as an adjunctive to INH/RIF antimicrobial therapy on monocyte profiling and atherosclerosis. Thus, we administered anti-CCR2 monoclonal antibody (clone AbAb-CCR2, Absolute Antibody®) by intraperitoneal injections twice weekly at a dose of 0.5 mg/kg for 8 weeks (16, 17), in conjunction with the 8 weeks of INH/RIF antimicrobial therapy (experimental weeks 4 to 12). Anti-CCR2 experimental intervention was conducted once was a proof-of-concept study. Mice on antimicrobial therapy alone but no anti-CCR2 injections served as a control group (Figure 3A).

Our primary endpoint was atherosclerotic plaque burden evaluated in *en face* aorta, and the secondary outcome was atherosclerotic plaque composition evaluated in cross-sectional aortic root, including plaque size and lipid lesions. TREM and CD68 plaque content as well as monocyte profiling were exploratory, mechanistic endpoints.

All procedures and animal care techniques were approved by the Institutional Animal Care and Use Committee of the University of Cincinnati and performed in accordance with guidelines and regulations of the National Institutes of Health U.S.A., and in compliance with ARRIVE guidelines.

### Atherosclerotic lesion assessment

We examined atherosclerotic lesions in aortic root sections and *en face* aorta harvested at time of euthanasia using Oil Red O staining. Sections fixed with 37% Formaldehyde (Fisher Scientific) were stained in Oil Red O working solution for 15 min. The sections were incubated with hematoxylin to counter stain. The sections were mounted on slides and images were taken after drying. Twelve frozen sections per mouse were examined throughout the aortic root and the average was considered for the results. Images were used to quantify the plaque area and lipid area using the Threshold method via ImageJ (NIH, Bethesda, MD). In addition, plaque cellular composition was assessed in aortic root sections. We used anti-F4/80 antibody at 1:100 dilution (rat IgG, ab6640, Abcam) to assess macrophage content, using VECTASTAIN® Elite® ABC-HRP Kit, Peroxidase (rat IgG, PK-6104, Vector Labs) and ImmPACT® DAB substrate kit, Peroxidase (SK-4105, Vector Labs). We also assessed triggering receptor expressed on myeloid cells 2 (TREM2^+^) content in atherosclerotic lesions with anti-TREM2 antibody at 1:125 dilution (goat IgG, A84373, antibodies.com), using VECTASTAIN® Elite® ABC-HRP kit, Peroxidase (goat IgG, PK-6105, Vector Labs) and ImmPACT® DAB substrate kit, Peroxidase (SK-4105, Vector Las). Images were captured using microscopy and the areas of positive brown stain per total area were estimated.

### Circulating lipids assessment

At the conclusion of the study, blood was collected from mice via intra-cardiac puncture immediately after euthanasia. To analyze the circulating lipids, plasma was separated from the blood. Total plasma cholesterol and triglyceride levels were measured using enzymatic assays (Infinity™ reagents).

### Immunophenotyping of circulating monocytes

Monocyte subsets were assessed using flow cytometry. The monocyte panel included CD45.2 (clone 104)/Brilliant Violet 711, CD11b (clone M1/70)/APC-Cy7, CD11c (clone HL3)/PECy7, Ly6G (clone 1A8)/PerCPCy5.5 and CD64 (clone X54-5/7.1)/Brilliant Violet 421 from BD Biosciences; CX_3_CR1 (clone SA011F11)/Brilliant Violet 510, CD36 (clone HM36)/Alexa Fluor 647, MHC-II (clone M5/115.15.2), and Ly6C (clone HK1.4)/Brilliant Violet 785 from Biolegend; CD115 (clone 61-1152-82)/PE-eflour610 from eBioscience; CD192 (CCR2) (clone 475301)/Alexa Fluor 700 from NOVUS. Flow cytometry data from the monocyte panels were acquired using a BD™ LSR II. Data were then analyzed using the FlowJo v10.10 software.

In addition, we tested the lipopolysaccharide (LPS) stimulation. Blood was mixed with an equal volume of RPMI medium containing LPS to a final concentration of 100 ng/mL and incubated at 37°C for 4 h. Following LPS stimulation, the plasma/medium supernatant was separated from the blood cells by centrifugation (2,500×g, 10 min). IL-6 secreted into the plasma/medium was quantified using IL-6 (M6000B) ELISA kit from R&D, according to manufacturer's instructions.

### Mycobacteria CFU enumeration

Right lungs and spleens were harvested at the time of euthanasia for enumeration of CFUs. Lung and spleen tissues were homogenized in 5 mL of sterile phosphate-buffered saline (PBS) and serially diluted on 7H10 agar (262710, BD Diagnostic) supplemented with 2.5 mg/L amphotericin B (A9528, Sigma), 26 mg/L polymyxin B sulfate (P4932-5MU, Sigma), 20 mg/L trimethoprim lactate (T0667-260 mg, Sigma), 50 mg/L carbenicillin disodium (C3416-1G, Sigma), and OADC enrichment (R450605, Fischer). CFUs were quantified following 2–3 weeks of humidified incubation at 37°C.

### Statistical analyses

Statistical analyses were carried out in Prism v10 and Stata v12 (College Station, TX). For numeric variables including flow cytometry data, we first assessed whether data were normally distributed using Shapiro–Wilk testing. For data with a normal distribution, t-test and ANOVA were used to compare two or multiple groups, respectively. For data without a normal distribution, we used Mann–Whitney-Wilcoxon and Kruskal–Wallis testing to compare two or multiple groups, respectively. Pairwise comparisons were performed when the overall test for multi-groups analysis was significant. *P*-values were adjusted for multiple comparisons using correction (Dunn's test). Statistical significance was considered based on *p* values <0.05. Although the execution of the experiments was not blinded, the statistical analyses for lesion quantification and flow cytometry were carried out in a blinded manner and calculation of all *p*-values values were two-tailed to minimize potential bias of a directional hypothesis. Experiments involving infected and uninfected controls and the effects of antimicrobial therapy were independently repeated. Data from these replicates were combined for analysis. Experiments involving anti-CCR2 treated mice and controls were performed once and were not independently repeated.

## Results

**Figure 2:**
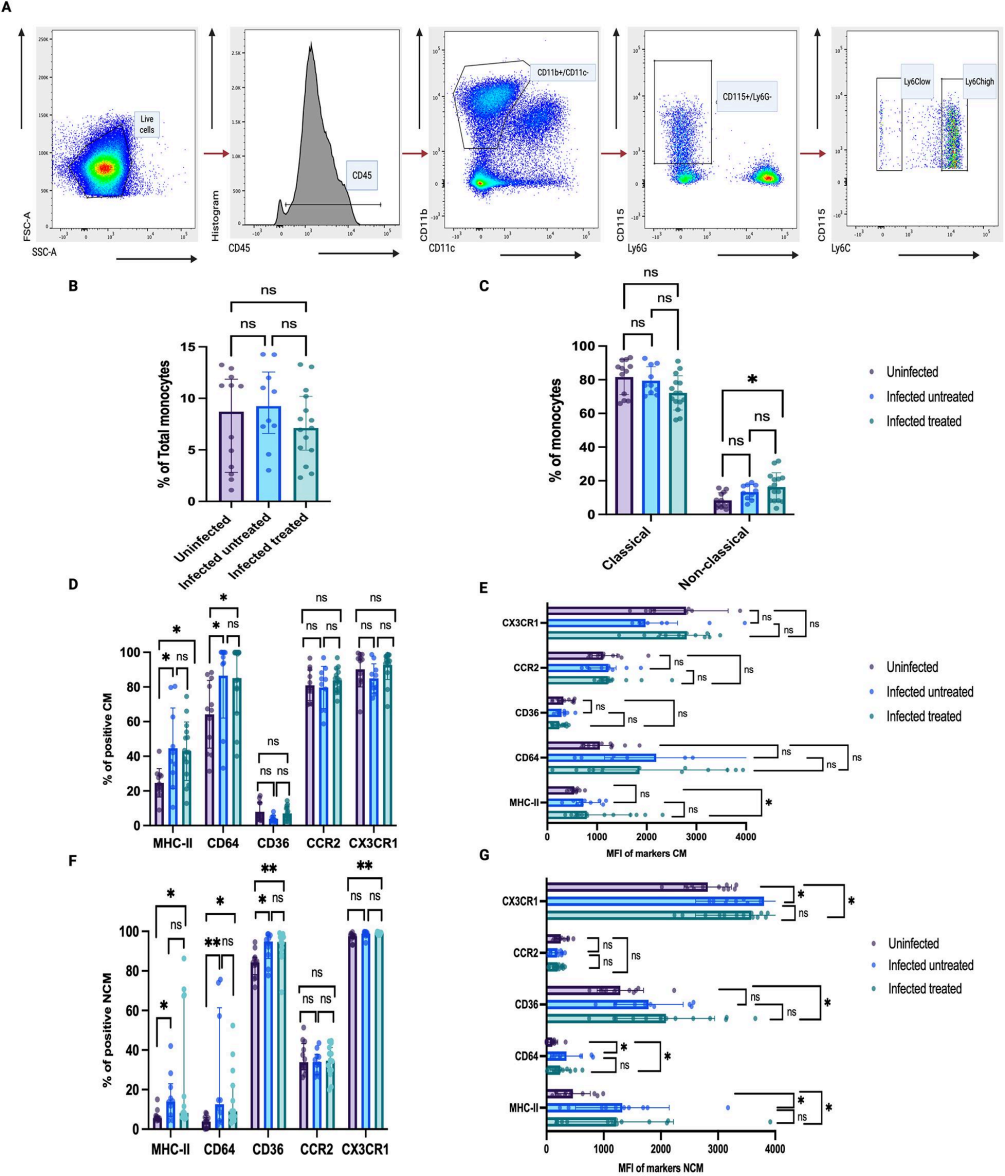
Antimicrobial therapy with rifampin and isoniazid did not reduce aggravated monocyte activation in mice infected with *M. bovis* BCG. Twelve-week-old male *Ldlr*^−/−^ mice were fed with western-type diet for up to 16 weeks. One randomly-selected group was used as controls (*n* = 12), while a second group was infected with *M. bovis* BCG. A group of infected mice were left untreated (*n* = 12), and the other group was treated with oral isoniazid and rifampin (INH/RIF) between weeks 4 and 12 (*n* = 16). At time of euthanasia (16 weeks), blood was obtained via cardiac puncture, and monocytes were profiled using flow cytometry. **(A)** Representative flow cytometry plots showing the gating strategy used to identify monocytes. Monocytes were defined as CD45+ CD3- CD11b + CD11c- CD115+ Ly6G- cells. Monocyte subsets were defined based on Ly6C expression. **(B)** Percentage of total monocytes across study groups. **(C)** Percentage of Ly6C^high^ classical monocytes and Ly6C^low^ non-classical monocytes across study groups. **(D)** Percentage of Ly6C^high^ classical monocytes positive for MHC-II, CD64, CD36, CCR2, CX3CR1 surface receptors. **(E)** Median fluorescence intensity (MFI) of MHC-II, CD64, CD36, CCR2, CX3CR1 surface receptors on Ly6C^high^ classical monocytes. **(F)** Percentage of Ly6C^low^ non-classical monocytes positive for MHC-II, CD64, CD36, CCR2, CX3CR1 surface receptors. **(G)** MFI of MHC-II, CD64, CD36, CCR2, CX3CR1 surface receptors on Ly6C^low^ non-classical monocytes. The experiment was independently performed twice and the data were combined. Each point represents the results in each mouse*.* Two mice from the infected untreated and 1 mouse from the infected treated were excluded. *P* values were the result of Kruskal–Wallis tests for multi-group analysis and Dunn's test for pairwise comparisons; *p*-values were adjusted for multiple comparisons; ns = no significant (*p* > 0.05); *p ≤ 0.05; ** *p* < 0.01; *** *p* < 0.001; **** *p* < 0.0001. Created in BioRender. Egoavil, R. (2026) https://BioRender.com/lgv0n4z.

INH/RIF antimicrobial therapy induced successful microbiologic clearance at 4 weeks post-antimicrobial treatment, as evidenced by negative cultures for mycobacteria from homogenized lung and spleen tissues at time of euthanasia, cultured in Mycobacteria specialized differential media. (Supplementary Figures S1A,B). In the BCG-infected control mice that did not receive INH/RIF antimicrobial therapy (untreated group), the median CFU/g in lung tissue was 1.313 × 10^5^ [interquartile range (IQR), 0.744 × 10^5^–1.917 × 10^5^] and the median CFU/g in spleen tissue was 0.155 × 10^5^ (IQR, 0.121 × 10^5^–0.235 × 10^5^) at the time of euthanasia (Supplementary Figures S1A,B). INH/RIF antimicrobial therapy had no significant effects on body weight nor plasma triglycerides or cholesterol levels, compared to control mice not receiving INH/RIF therapy (Supplementary Figures S2A–C). Two mice from the infected untreated group and one mouse from the infected treated group were excluded from experiments analyses involving infected and uninfected controls and the effects of antimicrobial therapy as it indicated in the corresponding figure legends (Figures 1, 2).

### Atherosclerosis burden remained aggravated after INH/RIF antimicrobial therapy

Compared to uninfected mice, BCG-infected mice and BCG-infected INH/RIF-treated mice exhibited an exacerbated atherosclerotic burden in *en face* aorta at 16 weeks [Area (µm): 266,909 vs. 548,990 vs. 483,570; *p* = 0.008; Figures 1A–D]. Plaque composition analysis showed that plaque size and lipid lesion areas were larger in the BCG-infected and BCG-infected INH/RIF treated mice compared to uninfected mice, as seen by the red tinction surrounding the aorta root in the infected groups, but the trends were not statistically significant (Figures 1C,E).

### Monocyte activation markers remained elevated after INH/RIF antimicrobial therapy

We used multi-dimensional flow cytometry to profile circulating monocytes (CD45+ CD3- CD11b + CD11c- CD115+ Ly6G- cells) (Figure 2A). The percentage of total circulating monocytes in blood was similar across groups (Figure 2B). Compared to uninfected mice, BCG-infected mice and BCG-infected INH/RIF-treated mice had an increased percentage of Ly6C^low^ non-classical monocytes (5.5% vs. 13.9% vs. 16.6%; *p* = 0.003) (Figure 2C) and increased expression of activation markers as CD64, CD36, MHC-II, CX3CR1 evaluated by percentage (Figure 2F) or receptor density with median fluorescence intensity (MFI) (Figure 2G). In Ly6C^high^ classical monocytes, we also observed increased density of the MHC-II receptor (Figure 2E) and increased percentage of MHC-II and CD64 receptors (Figure 2D).

### Addition of anti-CCR2 to INH/RIF antimicrobial therapy decreased atherosclerosis

**Figure 3:**
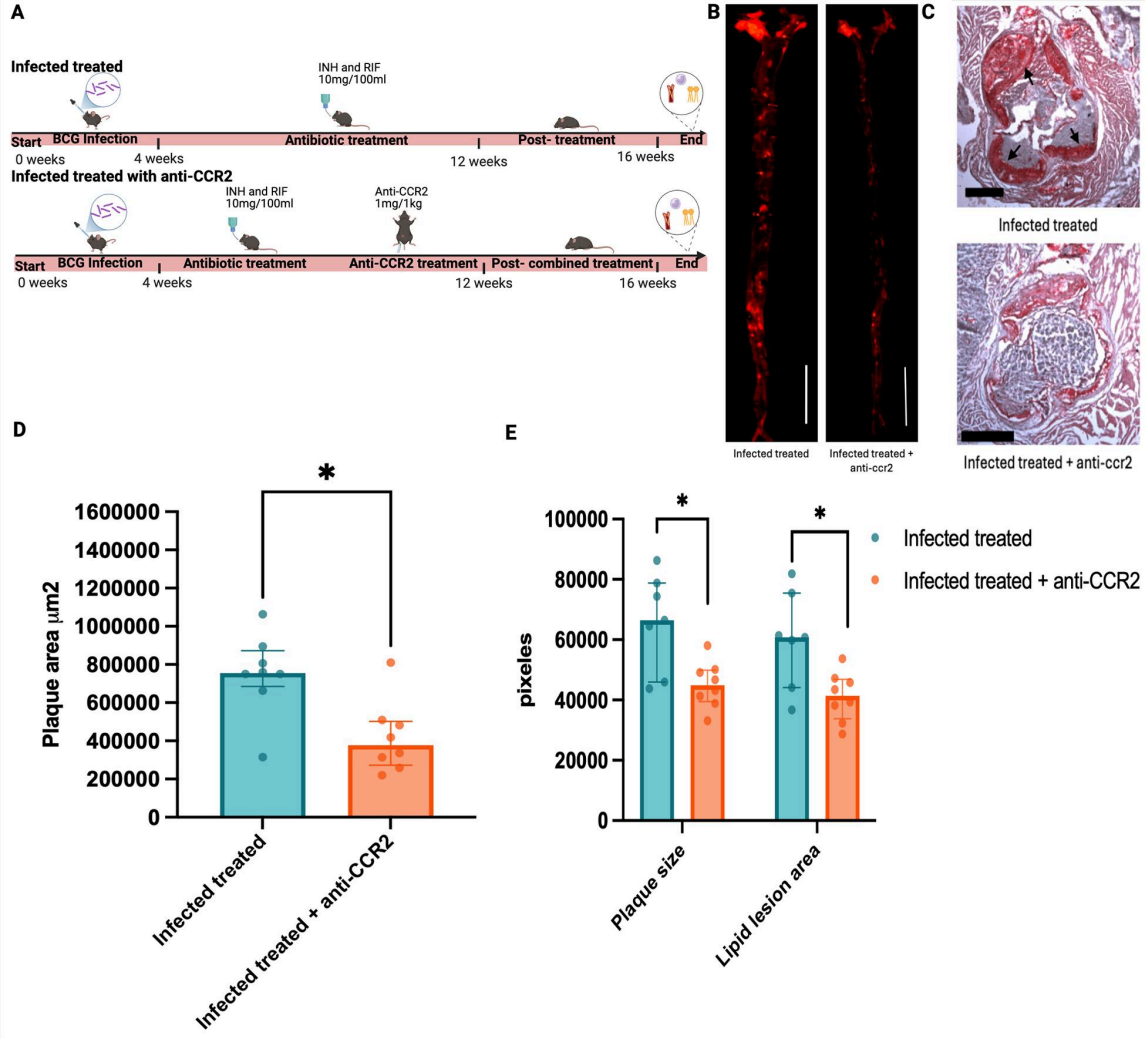
The addition of adjunctive anti-CCR2 to antimicrobial therapy decreased atherosclerosis development in mice infected with *M. bovis* BCG. **(A)** Twelve-week-old male *Ldlr*^−/−^ mice were infected with *M. bovis* BCG and then fed with western-type diet for 16 weeks. Mice were treated with oral isoniazid and rifampin (INH/RIF) between weeks 4 and 12 to clear the mycobacterial infection (*n* = 8). One randomly-selected group received anti-CCR2 adjunctive therapy in addition to INH/RIF during weeks 4 to 12 (*n* = 8). Created in BioRender. Egoavil, R. (2026) https://BioRender.com/tf0ru23. **(B)** Data representative of the aortae of one BCG-infected mouse treated with INH/RIF and one BCG-infected mouse treated with INH/RIF plus anti-CCR2 adjunctive therapy. Images were processed by ImageJ, with a magnification of 500 μm 10X. **(C)** Representative microscopic views of aortic root sections stained with Oil Red O, from one BCG-infected mouse treated with INH/RIF and one BCG-infected mouse treated with INH/RIF plus anti-CCR2 adjunctive therapy, with a magnification of 500*μ*m 4X 1 × 1 scale. **(D)** Atherosclerotic lesions in *en face* aorta were examined using Oil Red O staining at week 16 and the plaque area was quantified in µm^2^. **(E)** Plaque size and lipid lesion area in aorta were assessed in the aortic roots using Oil Red O staining. Data are from one experiment. Each point represents the results from one mouse*. P* values were the result of Mann–Whitney tests; ns = no significant (*p* > 0.05); *p ≤ 0.05; ** *p* < 0.01; *** *p* < 0.001; **** *p* < 0.0001.

We then investigated whether adjunctive anti-CCR2 treatment to antimicrobial therapy influenced infection-aggravated atherosclerosis (Figure 3A). Overall, the addition of twice weekly anti-CCR2 monoclonal antibody during INH/RIF antimicrobial therapy had no significant effects on body weight (Supplementary Figure S3A). We observed no significant differences in triglyceride and cholesterol levels at weeks 12 and 16 (Supplementary Figures S3B,C). Anti-CCR2 adjunctive therapy did not affect INH/RIF microbiologic clearance, which was confirmed through negative cultures for mycobacteria from lung and spleen tissues at time of euthanasia (4 weeks post-INH/RIF therapy; Supplementary Figures S1C,D).

We found that BCG-infected INH/RIF-treated mice that received adjunctive anti-CCR2 had significantly lower atherosclerosis burden in *en face* aorta, compared to BCG-infected INH/RIF-treated mice without anti-CCR2 adjunctive therapy (Figures 3B,D). Further, the atherosclerotic burden in *en face* aorta of BCG-infected INH/RIF-treated mice that received adjunctive anti-CCR2 was similar to the atherosclerotic burden of uninfected mice (Supplementary Figure S4), indicating that antimicrobial treatment with adjunctive anti-CCR2 therapy reduces the effects of infection-aggravated atherosclerosis in these proof-of-concept studies.

Atherosclerotic plaque composition analysis revealed that anti-CCR2 adjunctive therapy led to significant decreases in plaque size and lipid lesion content in aortic roots, compared to control mice noted by the absence of red areas surrounding the aortic root in opposition to the infected control (Figures 3C,E). Overall macrophage content within atherosclerotic lesions was assessed by F4/80 staining in aortic roots and corrected by the total amount of atherosclerotic plaque found in *en face* aorta. We found that F4/80 macrophage content had a trend toward reduction in the anti-CCR2 group compared to controls, although the trends were not statistically significant (*p* = 0.078; Supplementary Figure S5A). “Similarly, anti-CCR2 treatment showed a borderline trend toward a reduction of overall TREM2 + content in the aorta (*p* = 0.064; Supplementary Figure S5B)”.

### Addition of anti-CCR2 to INH/RIF antimicrobial therapy decreased monocyte activation markers

**Figure 4:**
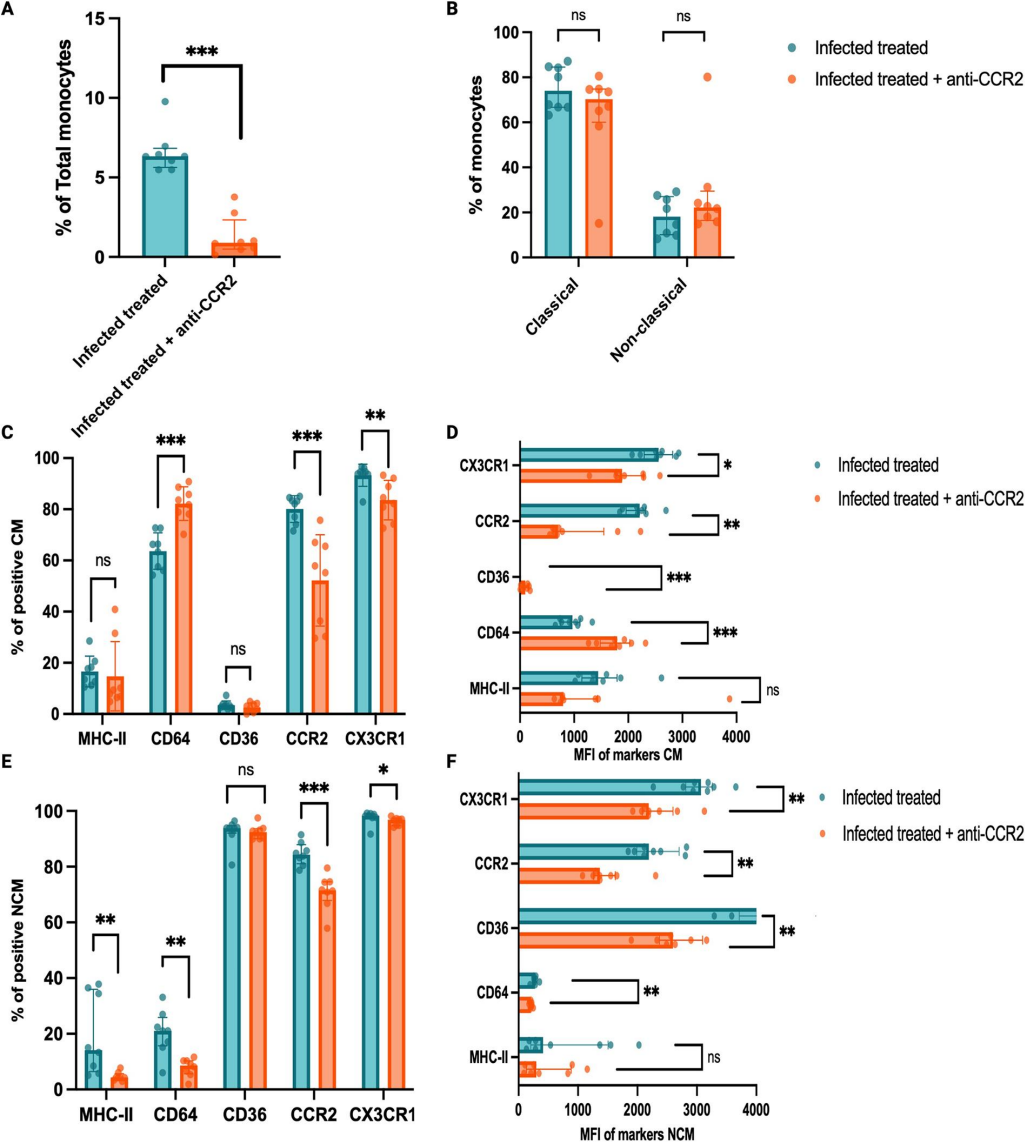
The addition of adjunctive anti-CCR2 to antimicrobial therapy decreased activation markers on monocytes in mice infected with M. bovis BCG. Twelve-week-old male *Ldlr*^−/−^ mice were infected with *M. bovis* BCG and then fed with western-type diet for 16 weeks. Mice were treated with oral isoniazid and rifampin (INH/RIF) between weeks 4 and 12 to clear the mycobacterial infection (*n* = 8). One randomly-selected group received anti-CCR2 adjunctive therapy in addition to INH/RIF during weeks 4 to 12 (*n* = 8). At time of euthanasia (16 weeks), blood was obtained via cardiac puncture, and monocytes were profiled using flow cytometry. **(A)** Percentage of total monocytes across study groups. **(B)** Percentage of Ly6C^high^ classical monocytes and Ly6C^low^ non-classical monocytes across study groups. **(C)** Percentage of Ly6C^high^ classical monocytes positive for MHC-II, CD64, CD36, CCR2, CX3CR1 surface receptors. **(D)** Median fluorescence intensity (MFI) of MHC-II, CD64, CD36, CCR2, CX3CR1 surface receptors on Ly6C^high^ classical monocytes. **(E)** Percentage of Ly6C^low^ non-classical monocytes positive for MHC-II, CD64, CD36, CCR2, CX3CR1 surface receptors. **(F)** MFI of MHC-II, CD64, CD36, CCR2, CX3CR1 surface receptors on Ly6C^low^ non-classical monocytes. Data are from one experiment. *P* values were the result of Mann–Whitney tests; ns = no significant (*p* > 0.05); *p ≤ 0.05; ** *p* < 0.01; *** *p* < 0.001; **** *p* < 0.0001. Created in BioRender. Egoavil, R. (2026) https://BioRender.com/t37d498.

BCG-infected INH/RIF-treated mice that received adjunctive anti-CCR2 treatments had a significant reduction on the percentage of total circulating monocytes, compared to BCG-infected INH/RIF-treated mice without anti-CCR2 adjunctive (Figure 4A), while the overall proportion of classical and non-classical monocyte subsets remained stable (Figure 4B). As expected, we observed a significant decline in the density of CCR2 on monocytes from mice treated with anti-CCR2 adjunctive therapy. At the monocyte subset level, anti-CCR2 treatment led to a reduction in the density of CX3CR1, CD36 and CD64 receptors on the surface of Ly6c^high^ classical monocytes by MFI (Figure 4D). Anti-CCR2 therapy also induced a decrease in density of CX3CR1, CD64, CD36 receptors on Ly6c^low^ non-classical monocytes by MFI (Figure 4F). We also observed decreased overall percentage of CX3CR1 + monocytes across classical and non-classical monocyte subsets (Figures 4C,E). After *ex vivo* lipopolysaccharide (LPS) stimulation of whole blood, we observed a significantly decreased production of interleukin-6 (IL-6) in mice treated with anti-CCR2 compared to control mice (Supplementary Figures S6A,B).

## Discussion

We found that monocyte activation and atherosclerosis burden remained elevated despite infection clearance with INH/RIF antimicrobial therapy, evaluated by cultures 4 weeks post-antimicrobial therapy. The addition of anti-CCR2 to antimicrobial therapy dampened monocyte activation and atherosclerosis development. Our results indicate that combining antimicrobials with CCR2 immunomodulation has a potential impact on ameliorating mycobacteria-aggravated atherosclerosis.

CCR2 is involved in the recruitment of monocyte/macrophages into tissues during physiological and pathological states. CCR2 is a CC-chemokine receptor with seven trans-membrane domains coupled to a guanosine triphosphate (GPT)-binding protein. Upon CCL2/MCP-1 (monocyte-chemoattractant protein 1) binding to CCR2, there is induction of intracellular protein kinases, inositol triphosphate formation, and release of calcium which facilitate cell activation and migration (18). Prior studies in the *apoE*^−/−^ mouse model have shown that complete absence of the CCR2 receptor (*CCR2*^−/−^, *apoE*^−/−^) results in decreased atherosclerotic lesion formation by inhibition of monocyte/macrophages recruitment into the vessel wall (12). Our findings are in line with these observations and expand into a protective role of CCR2 modulation as an adjunctive therapy to standard of care antimicrobials to decrease infection-aggravated atherosclerosis. It is important, nonetheless, to define the most appropriate timing and magnitude of CCL2/CCR2 axis modulation in future studies, since recruitment of monocytes and other immune cells through CCL2/CCR2 interactions is not only important in atherosclerosis development, but also relevant for preventing and controlling mycobacterial infections (19, 20). In our experiments, CCR2 modulation was used together with effective antimicrobial therapy, and we did not observe microbiologic failure based on cultures obtained at time of euthanasia (4 weeks post antimicrobial therapy completion). Future studies could investigate the delicate balance of CCL2/CCR2 axis modulation required to reduce infection-driven exacerbated monocyte activation and atherosclerosis, while minimizing the risk of infection treatment failure or relapse, which will be important to safely translate these promising findings into human trials.

Analyzing markers linked to inflammation due to infection, we found that anti-CCR2 adjunctive therapy decreased the expression of key activation markers on the surface of monocytes, including MHC-II, CD64, and CD36 on non-classical monocytes, as well as an overall reduction of IL-6 production from whole blood immune cells. Of interest, CD36 is a scavenger receptor responsible for cellular uptake of oxidized lipids and thus has an important role in macrophage foamy cell formation and atherosclerotic lesion development (21, 22). In our experiments, adjunctive anti-CCR2 therapy decreased the expression of CD36 on monocytes. Moreover, we observed decreased lipid content within atherosclerotic lesions of mice treated with adjunctive anti-CCR2 therapy. We also observed a trend toward decreased TREM2 + cellular content in aortae among mice treated with anti-CCR2 modulation. Of note, TREM2 is a cell surface lipid sensor that promotes foamy macrophage lipid uptake and survival in atherosclerosis (23). Taken together, our findings indicate that *in vivo* CCR2 modulation in the context of anti-mycobacterial treatment may exert anti-atherogenic properties not only by reducing recruitment of monocyte/macrophages into vessels, but also by shifting the phenotype and function of circulating monocytes towards a less pro-inflammatory profile, particularly the non-classical monocyte subset. Future studies can investigate the specific interactions between the CCR2/CCL2 axis and CD36 and TREM2 in the regulation of intracellular lipid abundance and metabolism.

Atherosclerosis is recognized as a complex, multifactorial inflammatory process (24). Infections may trigger inflammatory responses and thus contribute to atherosclerosis development, even when the nidus of primary infection is distant, such as in the lungs (2, 25, 26). Thus, pulmonary infections have been associated with increased risk of acute myocardial infarction, even several weeks after the infection has cleared (27). We previously demonstrated that *Mycobacterium bovis* Bacille-Calmette-Guérin, an attenuated strain of the *Mycobacterium tuberculosis* complex, enhances monocyte activation and atherosclerosis development (11). Patients affected by tuberculosis carry an approximately two-times increased likelihood of acute myocardial infarction compared to people without tuberculosis, independent of traditional cardiovascular risk factors (6, 7). Recently, we reported increased subclinical obstructive coronary atherosclerosis among people with tuberculosis infection compared to non-tuberculosis controls thus providing evidence in humans of increased atherosclerosis burden in the setting of tuberculosis infection (28).

Importantly, although tuberculosis is treatable with antimicrobials, multiple studies have shown that the risk of cardiovascular events and cardiovascular mortality remain elevated in people affected by tuberculosis disease, even after clinical and microbiological cure (8). Nevertheless, a common limitation when investigating the effects of infectious diseases in atherosclerosis within human populations is that cardiovascular risk can be affected by a myriad of individual, social, and environmental factors that are difficult to fully account for in epidemiological studies. Our experimental approach in the extensively validated *Ldlr*^−/−^ model of atherosclerosis allowed us to isolate the effect of mycobacterial disease in the atherosclerotic process and demonstrate that antimicrobial therapy alone is not sufficient to reduce mycobacteria-aggravated atherosclerosis. More importantly, our proof-of-concept experiments show that monocyte-driven inflammation and atherosclerosis can be modulated by adding anti-CCR2 as an adjunctive to antimicrobial therapy.

Our study had limitations. Whereas CCR2 is primarily involved in recruitment of monocytes into the vessels and atherosclerotic plaque, studies show that this receptor is also important for homing of T cells, particularly IFN-γ-producing T cells, that could impact plaque formation (29). Future studies of CCR2-modulating agents should include evaluation of both monocytes and T cells. We used differential media specialized for Mycobacteria to assess microbiologic clearance which was confirmed by complete absence of CFU colonies upon direct plate inspection. Future studies should use more sensitive methodology, including lung pathology and other bacterial burden surrogate markers, to confirm our findings throughout different timepoints post-CCR2 treatment, since CCR2 inhibition could affect susceptibility to tuberculosis and risk of post-treatment relapse. We measured CCR2 expression via flow cytometry using a fluorophore-conjugated anti-CCR2 antibody. Although flow cytometry experiments were conducted 4 weeks after the last anti-CCR2 therapeutic dose, it is possible that binding to residual anti-CCR2 therapeutic antibody could compete with the detection of CCR2 by flow cytometry and therefore falsely decrease CCR2 expression. However, this potential limitation in CCR2 detection does not impact the main atherosclerosis findings from the anti-CCR2 therapeutic experiments. Moreover, prior mouse studies have demonstrated that pharmacologic targeting of the CCR2/CCL2 axis can reduce plaque formation (17), but our pilot study adds novelty on the potential effects of CCR2 inhibition in reducing atherosclerosis within the post-infection context. Since the anti-CCR2 intervention was performed as a single proof-of-concept experiment, further studies are warranted to replicate anti-CCR2 modulation findings within a larger sample population,under different dosing strategies and timepoints relative to infection and antimicrobial therapy before considering translation of these preliminary findings into human studies.

## Conclusion

We found that monocyte activation and atherosclerosis burden remained elevated despite mycobacterial clearance with INH/RIF antimicrobial therapy. The addition of anti-CCR2 to antimicrobial therapy dampened monocyte activation and atherosclerosis development. Our results indicate that combining antimicrobials with CCR2 immunomodulation may reduce infection-aggravated atherosclerosis.

## Data Availability

The raw data supporting the conclusions of this article will be made available by the authors, without undue reservation.
