# Supplementary material for: Antimicrobial therapy combined with C-C chemokine receptor type 2 modulation dampens mycobacteria-aggravated monocyte activation and atherosclerosis
Source: Front Cardiovasc Med. 2026 Mar 31;13:1753414. doi: 10.3389/fcvm.2026.1753414 (PMC13076342; doi:10.3389/fcvm.2026.1753414)

SUPPLEMENTARY FIGURES

Supplement to: Antimicrobial Therapy Combined with CCR2 Modulation Dampens Mycobacteria-Aggravated Monocyte Activation and Atherosclerosis

Supplementary Figure 1. Colony forming units (CFU) counts from homogenized lungs and spleens in PBS, cultured in differential media for *M. tuberculosis*, confirming microbiologic clearance in the groups of experiments involving infected and uninfected controls and the effects of antimicrobial therapy. Two mice from the infected untreated and 1 mouse from the infected treated were excluded. P values were the result of Kruskal-Wallis tests for multi-group analysis and Dunn's test for pairwise comparisons; p-values were adjusted for multiple comparisons (A). Negative cultures confirming microbiological clearance in the groups of experiments involving the adjunctive anti-ccr2 therapy. *P* values were the result of Mann-Whitney tests (B) ns = no significant ($p>0.05$); *$p \leq 0.05$; ** $p<0.01$; *** $p<0.001$; **** $p<0.0001$.

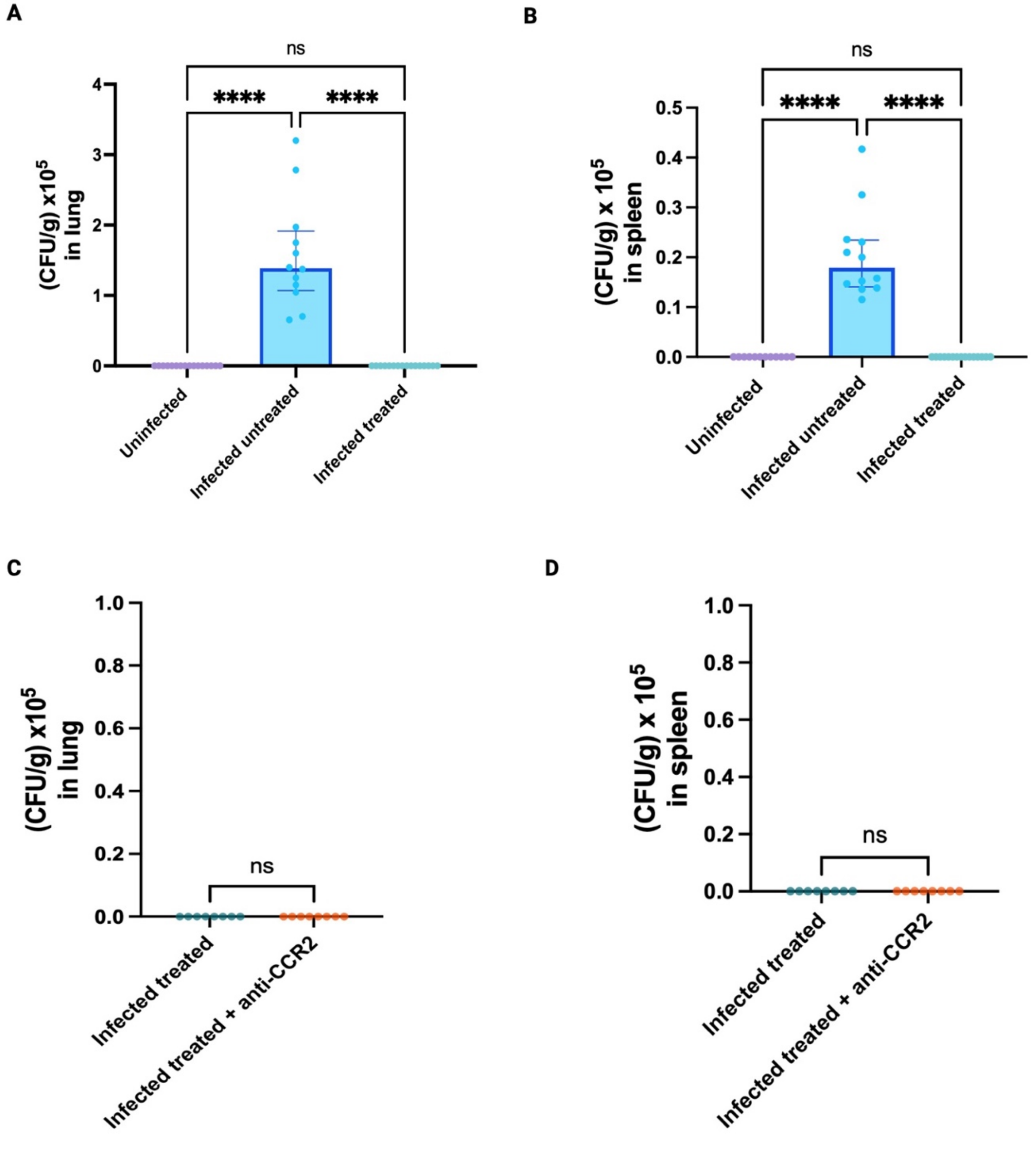

A
ns
****
****
4
3
2
1
0
(CFU/g) x10^5 in lung
Uninfected
Infected untreated
Infected treated
B
ns
****
****
0.5
0.4
0.3
0.2
0.1
0.0
(CFU/g) x 10^5 in spleen
Uninfected
Infected untreated
Infected treated
C
1.0
0.8
0.6
0.4
0.2
0.0
(CFU/g) x10^5 in lung
ns
Infected treated
Infected treated + anti-CCR2
D
1.0
0.8
0.6
0.4
0.2
0.0
(CFU/g) x 10^5 in spleen
ns
Infected treated
Infected treated + anti-CCR2

Supplementary Figure 2. Comparison of **(A)** body weight (in grams), and **(B)** triglycerides and **(C)** total cholesterol plasma levels (in mg/L) of uninfected mice, BCG-infected mice left untreated, and BCG-infected mice treated with isoniazid/rifampin antimicrobial therapy. All measurements were obtained by week 16 at time of euthanasia. The experiment was independently performed twice and the data were combined. Each point represents the results in each mouse. Two mice from the infected untreated and 1 mouse from the infected treated were excluded. *P* values were the result of Kruskal-Wallis tests for multi-group analysis and Dunn's test for pairwise comparisons; p-values were adjusted for multiple comparisons; ns = no significant (p>0.05); **p* ≤ 0.05; ** *p*<0.01; *** *p*<0.001; **** *p*<0.0001.

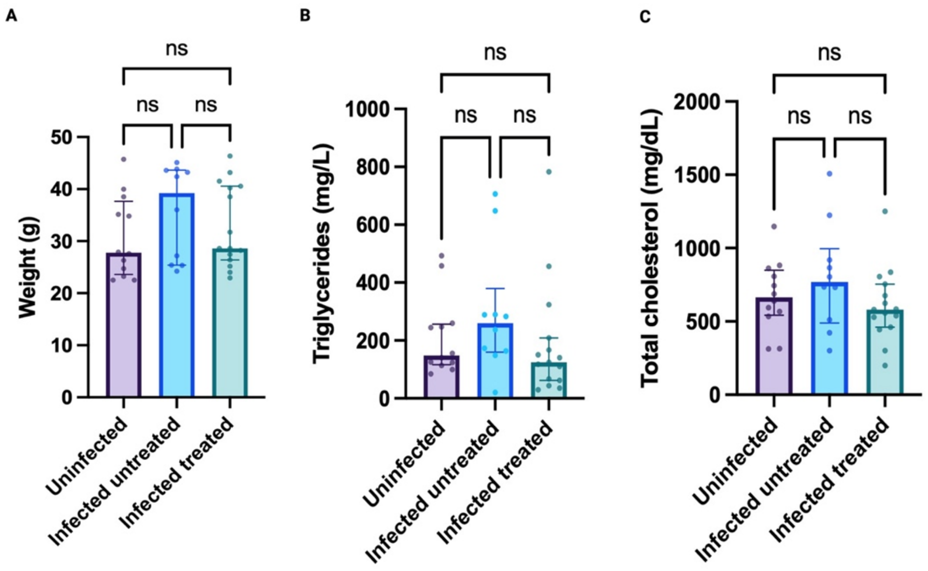

Supplementary Figure 3. Comparison of **(A)** body weight (in grams), and **(B)** triglycerides and **(C)** total cholesterol plasma levels (in mg/L) of mice infected with *Mycobacterium bovis* BCG treated with isoniazid/rifampin antimicrobials, and mice treated with isoniazid/rifampin antimicrobials plus anti-CCR2 adjunctive therapy. *P* values were the result of Mann-Whitney tests; ns = no significant (p>0.05); *p ≤ 0.05; ** p<0.01; *** p<0.001; **** *p*<0.0001.

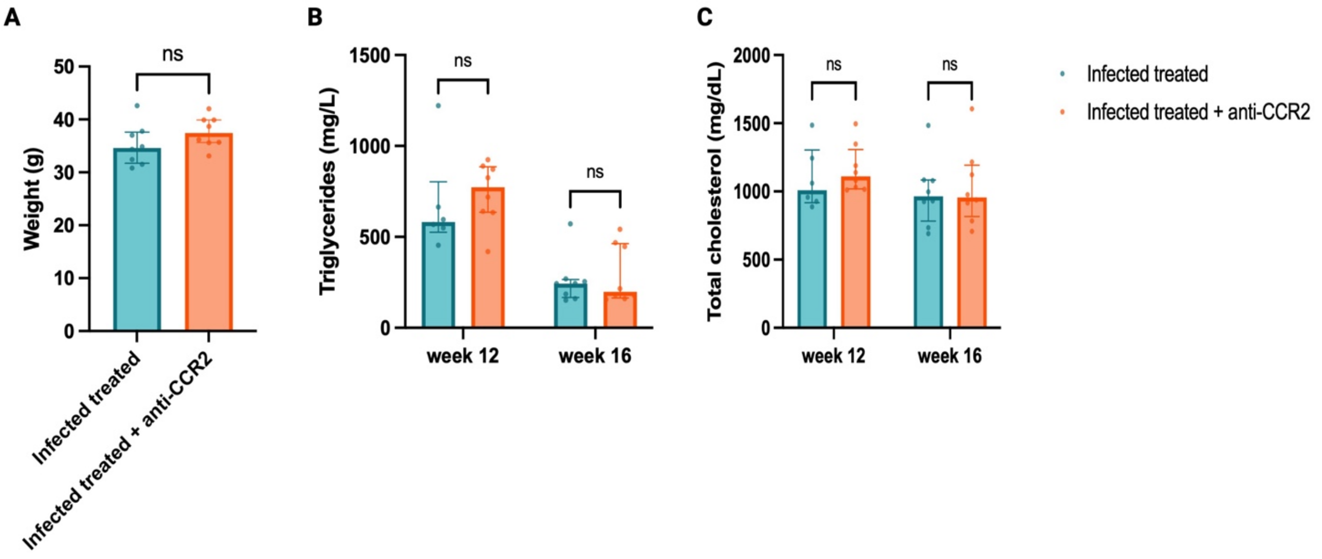

Supplementary Figure 4. Comparison between the plaque area (in μm^2) evaluated in *en face* aorta assessed in the groups of BCG-infected mice treated with isoniazid/rifampin vs. BCG-infected mice treated with isoniazid/rifampin plus anti-CCR2 adjunctive therapy

P values were the result of Mann-Whitney tests; ns = no significant ($p>0.05$); *$p \leq 0.05$; ** $p<0.01$; *** $p<0.001$; **** $p<0.0001$.

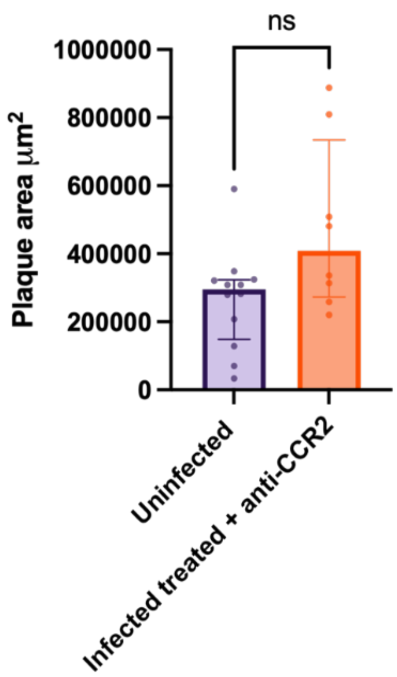

Supplementary Figure 5. Total quantification on *en face* aorta for **(A)** F4/80+ and **(B)** TREM2+ cellular content, in mice infected with *Mycobacterium bovis* BCG and treated with isoniazid/rifampin antimicrobials, and mice infected with *Mycobacterium bovis* BCG treated with isoniazid/rifampin plus anti-CCR2 adjunctive therapy. *P* values were the result of Mann-Whitney tests; ns = no significant (p>0.05); *p ≤ 0.05; ** p<0.01; *** p<0.001: **** *p*<0.0001.

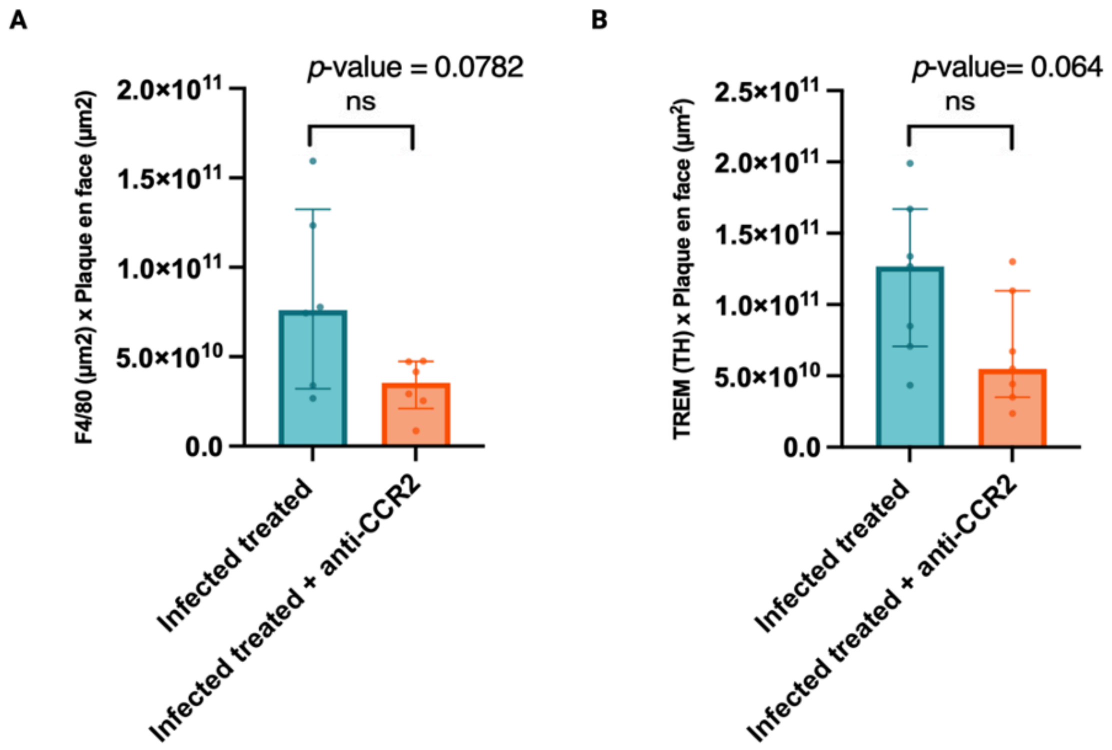

Supplementary Figure 6. (A)Interleukin (IL)-6 production (in pg/mL) from circulating immune cells after 6-hours stimulation with lipopolysaccharide (LPS); **(B)** and the production of IL-6 per number of leukocytes ($CD45^{+}$) were assessed in the groups of BCG-infected mice treated with isoniazid/rifampin vs. BCG-infected mice treated with isoniazid/rifampin plus anti-CCR2 adjunctive therapy. *P* values were the result of Mann-Whitney tests; ns = no significant ($p>0.05$); *$p \leq 0.05$; ** $p<0.01$; *** $p<0.001$; **** $p<0.0001$

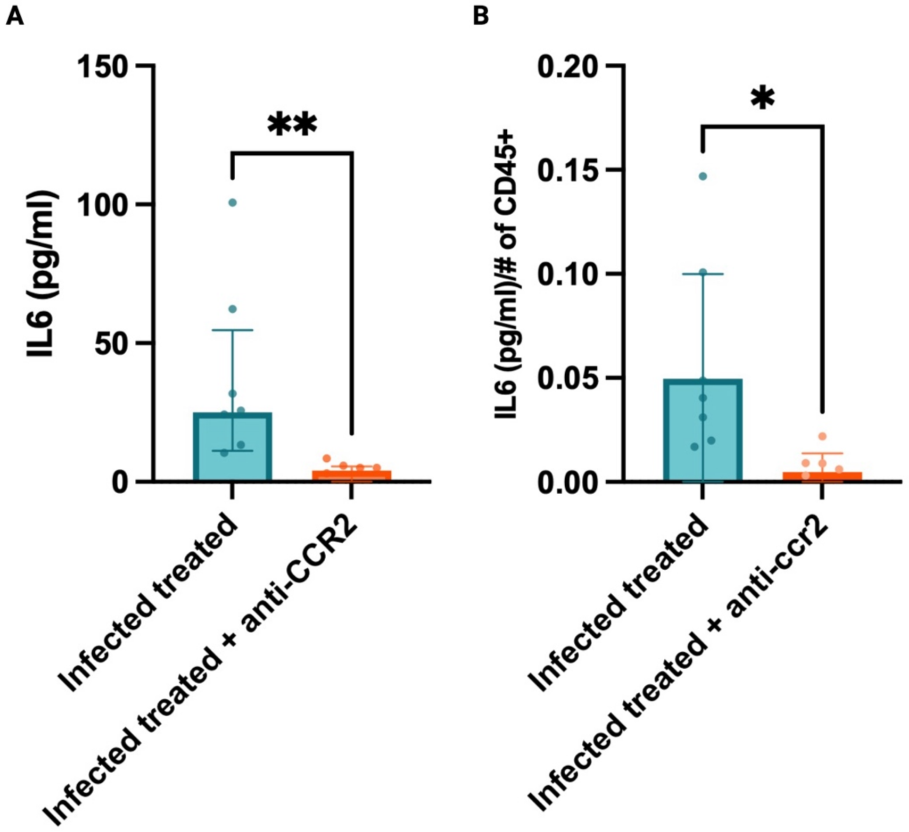

Supplement: Supplementary file 1 [file Datasheet1.pdf]
